# A Systematic Review and Meta-analysis of Sex Differences in Subcutaneous and Visceral Abdominal Fat in Children

**DOI:** 10.1093/nutrit/nuaf143

**Published:** 2025-08-11

**Authors:** Jose Guillermo Ortega-Avila, Alejandro Segura Ordoñez, Harry García Muñoz, Milton Fabian Suarez Ortegon, Blanca C Salazar Contreras

**Affiliations:** Departamento de Ciencias Básicas de la Salud, Facultad Ciencias de la Salud, Pontificia Universidad Javeriana Seccional Cali, 760031 Cali, Colombia; Grupo de investigación Salud y Movimiento, Facultad de Salud, Universidad Santiago de Cali, 760042 Cali, Colombia; Grupo de Nutrición, Departamento de Ciencias Fisiológicas, Facultad de Salud, Universidad del Valle, 760043 Cali, Colombia; Grupo de investigación Salud y Movimiento, Facultad de Salud, Universidad Santiago de Cali, 760042 Cali, Colombia; Grupo de Nutrición, Departamento de Ciencias Fisiológicas, Facultad de Salud, Universidad del Valle, 760043 Cali, Colombia; Grupo de Investigación SportScience, INDERVALLE, 760033 Cali, Colombia; Facultad de Ciencias de la Salud, Universidad Icesi, Cali 760031, Colombia; Departamento de Alimentación y Nutrición, Facultad de Ciencias de la Salud, Pontificia Universidad Javeriana Seccional Cali, 760031 Cali, Colombia; Grupo de Nutrición, Departamento de Ciencias Fisiológicas, Facultad de Salud, Universidad del Valle, 760043 Cali, Colombia

**Keywords:** subcutaneous abdominal adipose tissue, visceral adipose tissue, prepuberty, children, sex, normal-weight

## Abstract

**Context:**

Sex-based differences in abdominal fat distribution are well documented in adults, with men typically accumulating more visceral adipose tissue (VAT), located around intra-abdominal organs, and women exhibiting higher levels of subcutaneous abdominal adipose tissue (SAAT), distributed in the abdominal subcutaneous layer. However, the developmental onset of these differences remains unclear.

**Objective:**

This systematic review and meta-analysis examined sex-specific VAT and SAAT distribution differences among normal-weight prepubertal children aged 0–10 years.

**Data Sources:**

A systematic search of PubMed, Embase, LILACS, and Web of Science was conducted to identify studies published from the inception of each database through June 2024.

**Data Analysis:**

Standardized mean differences (SMDs) for sex-based differences in VAT and SAAT were calculated using a random-effects model, stratified by age group. The analysis included data from 20 studies. In the overall analysis, prepubertal girls had significantly higher SAAT compared with boys (pooled SMD = 0.23; 95% CI: 0.15–0.32; I^2^ = 89%). This sex difference became apparent starting in the 3- to 6-year age group (SMD = 0.59; 95% CI: 0.38–0.79; I^2^ = 80%). In contrast, no significant sex differences in VAT were detected in either the age-stratified analyses or the overall estimate (pooled SMD = 0.09; 95% CI: –0.01 to 0.19; I^2^ = 91%). However, this finding should be interpreted with caution due to the high degree of heterogeneity observed across studies.

**Conclusion:**

Sex differences in abdominal adiposity appear to emerge early in childhood, with prepubertal girls exhibiting higher SAAT than boys from the age of 3 years. In contrast, the absence of sex-based differences in VAT should be interpreted with caution.

**Systematic Review Registration:**

PROSPERO registration no. CRD42022361412.

## INTRODUCTION

Abdominal adipose tissue, based on anatomical localization, is classified into visceral adipose tissue (VAT), which surrounds the intra-abdominal organs, and subcutaneous abdominal adipose tissue (SAAT).^1^ In adulthood, men typically have higher VAT depots, whereas women during reproductive age have greater total fat mass and more fat in the subcutaneous compartment, including SAAT,^2–4^ with these sex differences reported to emerge during puberty.^5^

Differences in the accumulation and distribution of SAAT and VAT in normal-weight adults have mainly been attributed to hormonal factors, with sex steroid hormones (estrogens and testosterone) being crucial regulators of regional fat-deposition patterns.^6–8^ Estrogens can modulate lipolysis and lipogenesis through adipose tissue estrogen receptors, promoting adipose expansion while inhibiting VAT storage.^9^ In contrast, testosterone suppresses adipocyte lipid uptake and reduces gluteofemoral fat deposition.^10^ An excessive fat accumulation in both VAT and SAAT has been associated with an increased risk of metabolic dysregulation in both childhood and adulthood,^11–16^ and these associations have shown variations according to the type of abdominal fat depots and sex.^17^^,^^18^

However, it is still unclear when, during development, sex differences in VAT and SAAT first appear. Variations in study design, measurement methods, and biological diversity may contribute to this uncertainty. Furthermore, differences by sex in indicators of adiposity, such as the total percentage of body fat and circulating levels of leptin, have been reported in children younger than 10 years,^19^ suggesting that sex-specific differences in abdominal fat distribution could emerge early during childhood. Prepubertal sex differences in adipose tissue distribution may arise from nonhormonal mechanisms, including chromosomal effects (XX vs XY differences in X-linked genes)^20^ and epigenetic regulation through sex-specific DNA methylation patterns.^21^

Although several studies have explored the overall adiposity differences in childhood, none have systematically examined how abdominal adiposity varies by sex before puberty. To explore this question, we conducted a systematic review and meta-analysis focusing on sex-specific patterns of VAT and SAAT among normal-weight prepubertal children aged 0–10 years.

## METHODS

The systematic literature search was conducted and documented following the Preferred Reporting Items for Systematic Reviews and Meta-Analyses (PRISMA) guidelines. The study protocol was registered at the International Prospective Register of Systematic Reviews (PROSPERO) under registration number CRD42022361412.

### Selection Criteria

The eligibility criteria for studies were established according to the PICOS (Population, Intervention, Comparison, Outcomes, and Study Design) criteria (Table 1). Both randomized and nonrandomized trials, as well as observational studies, were eligible for inclusion in the systematic review.

**Table 1.** PICOS Criteria for Inclusion of Studies

Parameter	Criteria
Population	Normal-weight prepubertal children aged 0–10 years
Intervention/exposure	Sex (masculine and feminine)
Comparison	Boys vs girls
Outcome	Differences in visceral adipose tissue and subcutaneous abdominal adipose tissue
Study design	Randomized and nonrandomized trials, observational cohort, and cross-sectional studies

Articles were included if SAAT and VAT were assessed using imaging techniques such as magnetic resonance imaging (MRI), computed tomography (CT), dual-energy X-ray absorptiometry (DXA), or ultrasonography, as these techniques are considered the most accurate for evaluating abdominal fat.^22^ To minimize the potential confounding effects of pubertal hormones on SAAT and VAT measurements, we restricted our analysis to normal-weight children aged 10 years or younger. This upper age limit precedes typical pubertal onset (Tanner stage II) in both sexes.^23^ Normal-weight was defined using body mass index (BMI) within 1 SD for age. When the BMI *z*-score (BMIz) was available, we used BMIz ±1 SD as the criterion. The most comprehensive study was selected when several reports were published using the same data. We excluded reports of case studies, conference abstracts, letters to the editor, review articles, and articles with incomplete/missing data. After eliminating duplicates, the retrieved articles from the databases were transferred to the online Rayyan software (Ouzzani et al, Cambridge, MA, USA),^24^ then 2 investigators independently screened articles by title and abstract according to predefined eligibility criteria. Discrepancies were resolved by consensus between the pair; otherwise, disagreements were resolved by a third researcher.

### Search Strategy

A comprehensive literature search was conducted in PubMed, Embase, LILACS, and Web of Science databases, without publication date, type of article, or language restrictions (Table 2). The search covered the inception of each database until June 31, 2024. The search strategy was developed using Medical Subject Headings (MeSH) and key words related to VAT and SAAT, including terms such as “adipose tissue,” “fat body,” “fat mass,” “body fat distribution” “abdominal fat distribution,” “body adiposity index,” “subcutaneous fat,” “subcutaneous abdominal fat,” “abdominal fat,” “abdominal visceral fat,” “intraabdominal adipose tissue,” “retroperitoneal adipose tissue,” “retroperitoneal fat,” “visceral adipose tissue,” “visceral fat,” “subcutaneous fat, abdominal,” “Intra-Abdominal Fat,” “abdominal adipose tissue,” “preperitoneal fat,” “trunk fat,” “sex,” “sex factors,” “gender,” “sex characteristics,” “gender differences,” “gender dimorphism,” “sex dimorphism,” “male,” “female,” “child,” “children,” “newborn,” “infant,” and “neonate.” Detailed search strategies are provided in the supplementary material (Table S1).

**Table 2.** Summary of Search Strategy

Database	Search terms	Limits/filters
PubMed	Terms related to adipose tissue (eg, *“*visceral fat,” “subcutaneous fat”), sex/gender (eg, “sex factors,” “gender differences”), and pediatric population	Humans; no language or date restrictions
Embase	Combination of Emtree terms and free-text for abdominal fat, sex/gender, and children	Humans; no language or date restrictions
Web of Science	Key words related to adipose tissue, sex differences, and pediatric age group	No language or date restrictions
LILACS	DeCS (Health Sciences Descriptors) terms related to abdominal fat, sex characteristics, and child age	Languages: English, Spanish, Portuguese

### Data Extraction and Risk-of-Bias Assessment

Data extraction was performed independently using a standardized, predesigned electronic form. The following data were extracted: authors, year of publication, country, design of the study, age of children, sample size (total, boys and girls), indicator of normal-weight (BMI or BMIz score), measurement of primary outcome VAT and SAAT (volume, area, thickness), and technique used to assess the adipose tissue. If studies reported both superficial and deep SAAT, we used the former for the meta-analysis. In cases where more than 1 eligible effect measure was available, we extracted the most comprehensive one (eg, volume over area or diameter, and area over diameter).

### Assessments of Risk of Bias and Study Quality

Two reviewers independently evaluated the methodological quality of all included studies. For observational studies (cohort and cross-sectional designs), we used the Newcastle-Ottawa Scale. This tool assesses the selection of the study groups, their comparability, and the ascertainment of either the exposure or outcome.^25^ A modified version of the Newcastle-Ottawa Scale was adapted for cross-sectional studies.^26^ Studies scoring 6 or higher were classified as high quality.

### Data Synthesis and Statistical Analysis

To evaluate the effect of sex on SAAT and VAT in children, we used measurements of volume, area, or diameter as indicators of adiposity. When the adipose mass was reported, it was converted to volume, assuming an adipose tissue density of 0.94 g/mL.^27^ If multiple techniques were used to measure the outcomes in the same study, we selected the gold-standard method (MRI or CT). For studies that did not report the mean and SD, these values were estimated using the method described by McGrath et al,^28^ provided that sufficient data were available. Additionally, subgroups within studies that shared the same age range and showed comparable results for SAAT and VAT (*P* > .05) were combined to enhance the precision of the analysis.

We conducted age-stratified analyses across 5 developmental periods: 0–4 months, 6–12 months, >1–2.9 years, 3–6 years, and >6–10 years, based on the mean or median age reported in each study. Given that the included studies measured adiposity using different units (volume, area, or thickness) and methodologies, we calculated effect sizes for SAAT and VAT using standardized mean differences (SMDs). Hedges' *g* was used to adjust the SMDs to reduce potential bias due to small sample sizes.^29^ Pooled SMDs were computed with a random-effects model using restricted maximum likelihood, and the CIs were adjusted using the Knapp-Hartung method.^30^ A random-effects model was chosen because we anticipated substantial heterogeneity across the included studies. This model accounts for both within-study and between-study variability, yielding a more conservative and generalizable estimate of the overall effect size. The magnitude of effect sizes was interpreted using conventional Cohen's thresholds for SMDs, where values less than 0.2 indicate small effects, 0.2–0.8 represent moderate effects, and those greater than 0.8 correspond to large effects.^31^ Publication bias was evaluated through funnel plots and Egger's regression when the number of studies was 6 or more.^32^^,^^33^ The *I^2^* statistic was used to assess the heterogeneity of the study outcomes and interpreted as non-heterogeneity (*I^2^* =0% to 30%), moderate heterogeneity (*I^2^* = 30% to 49%), substantial heterogeneity (*I^2^* = 50% to 74%), and considerable heterogeneity (*I^2^* = 75% to 100%).^34^ The sensitivity analysis was performed sequentially, excluding 1 study at a time.^35^ Subgroup analyses, along with univariate meta-regression, were performed to investigate potential sources of heterogeneity. Factors examined included age, study design, measurement technique, geographic region, and study quality. The analysis was performed in the R programming language with the R-studio platform version 4.0.2 (R Foundation for Statistical Computing, Vienna, Austria)^36^ using Meta^37^ and Estmeansd packages. A *P* value less than or equal to .05 was considered statistically significant for all analyses. Bonferroni correction was applied to address multiple comparisons in subgroup analyses, adjusting the significance based on the number of comparisons performed.

## RESULTS

### Study Identification and Selection

**Figure 1.**
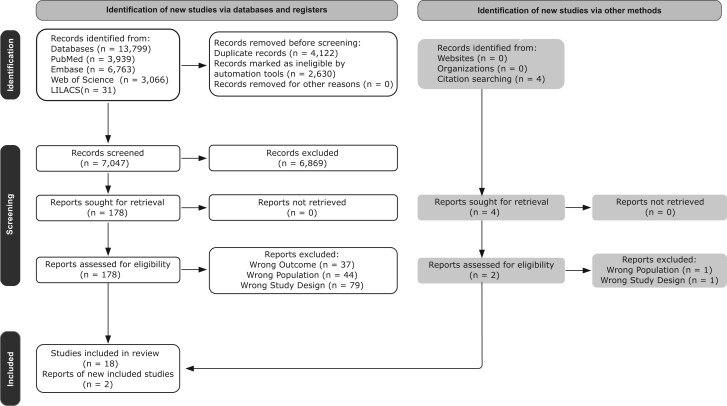
PRISMA (Preferred Reporting Items for Systematic Reviews and Meta-Analyses) Flowchart of the Study Literature Search and Inclusion for the Systematic Review and Meta-analysis to Determine Sex Differences in SAAT and VAT. Abbreviations: SAAT, subcutaneous abdominal adipose tissue; VAT, visceral adipose tissue

In the initial search, 13 799 articles were identified. After excluding 6752 articles, 7047 were screened based on their title and abstracts. Of these, 6869 were excluded because they did not meet the eligibility criteria. We conducted a thorough review of the full-text content of 178 articles. Of these, 18 articles met our inclusion criteria and 2 additional articles were included by citation searching. A total of 20 articles^38–57^ were included in the systematic review and meta-analysis (Figure 1).

### Characteristics of Studies

The studies included in the meta-analysis were published between 1997 and 2020. Of these, 13 studies (65%) were cohort studies,^38^^,^^40–48^^,^^50^^,^^56^^,^^57^ which reported 29 publications of SAAT and VAT, and 7 articles (35%) were from cross-sectional studies,^39^^,^^49^^,^^51–55^ which reported 9 records of abdominal fat. Ten studies (50%) were conducted in European populations,^38^^,^^40^^,^^41^^,^^43–46^^,^^48^^,^^53^^,^^56^ 8 studies (40%) were in the Americas,^39^^,^^42^^,^^49–52^^,^^55^^,^^56^ and 2 studies (10%) were from Asia^47^^,^^54^ (Table 3). After the assessment of the methodological quality, 4 studies were classified as low quality and 16 studies were classified as high quality (Table S2).

**Table 3.** Characteristics of Selected Studies

Studies by age group (year)	Design	Country	Age		BMI/BMI z-score/BMI SDS/weight		SAAT						VAT					
			Girls	Boys	Girls	Boys	n	Girls	n	Boys	Units	Measurement	n	Girls	n	Boys	Units	Measurement
0–4 months																		
De Lucia Rolfe et al (2013)^38^	Cohort	United Kingdom	3 mo		5.8 (0.7)^a^	6.4 (0.83)^a^	233	0.4 (0.1)^b^	254	0.4 (0.1)^b^	cm	US	233	2.3 (0.6)^b^	254	2.3 (0.6)^b^	cm	US
Ferreira et al (2014)^39^	Cross-sectional	Brazil	20 d		3332 (517)^c^	3326 (424)^c^	44	0.22 (0.04)^b^	55	0.20 (0.05)^b^	cm	US	44	5.2 (0.9)^b^	55	5.0 (0.8)^b^	cm	US
Brei et al (2015)^40^	Cohort	Germany	6 wk		15.2 (1.3)^c^	15.4 (1.2)^c^	76	33.0 (11.9)^b^	84	28.8 (12.2)^b^	mm^2^	US	74	10.9 (3.9)^b^	76	10.6 (3.2)^b^	mm^2^	US
			4 mo		16.1 (1.3)	16.6 (1.3)	77	44.2 (14.8)^b^	80	38.8 (14.9)^b^			79	13.3 (3.9)^b^	76	12.7 (4.2)^b^		
Gale et al (2015)^41^	Cohort	United Kingdom	13 d	13 d	3546 (0.51)^c^	3637 (0.489)^c^	29	0.1 (0.08; 0.013)^d^	40	0.09 (0.07; 0.12)^d^	L	MRI	29	0.017 (0.01; 0.02)^d^	40	0.017 (0.01; 0.02)^d^	L	MRI
			62 d	64 d	5219 (0.533)	5492 (0.687)	30	0.25 (0.22; 0.32)^d^	39	0.25 (0.19; 0.31)^d^			30	0.03 (0.02; 0.04)^d^	39	0.03 (0.02; 0.03)^d^		
Barros et al (2016)^42^	Cohort	Brazil	1 mo		3346 (479)^c^	3345 (508)^c^	11	29 (10)^b^	5	27.3 (10.5)^b^	mm^3^	US	11	9.9 (2.2)^b^	5	9.6 (2.8)^b^	mm^3^	US
					3005 (649)^c^	3205 (438)^c^	19	27.1 (10.7)^b^	18	28.8 (10.4)^b^			19	10.4 (2.8)^b^	18	10.2 (2.9)^b^		
					3134 (679)^c^	3115 (433)^c^	11	29.3 (10.5)^b^	12	23.3 (10.3)^b^			11	10.7 (2)^b^	12	10.5 (2.1)^b^		
			3 months		7144 (538)^c^	6900 (554)^c^	11	55.3 (10.1)^b^	5	48.1 (11.5)^b^			11	13.8 (1.7)^b^	5	14.2 (1.9)^b^		
					6329 (921)^c^	6630 (635)^c^	19	53.3 (10.6)^b^	18	52 (10.9)^b^			19	14.1 (1.8)^b^	18	13.8 (2.3)^b^		
					6076 (437)^c^	6667 (719)^c^	11	48.8 (10.3)^b^	12	50 (10.6)^b^			11	13.7 (1.5)^b^	12	12.4 (2)^b^		
de Fluiter et al (2020)^43^	Cohort	NL	3 mo		5.69 (0.70)^a^	6.2 (0.67)^a^	173	0.40 (0.12)^b^	228	0.41 (0.11)^b^	cm	US	173	2.34 (0.59)^b^	228	2.46 (0.63)^b^	cm	US
6–12 months																		
De Lucia Rolfe et al (2013)^38^	Cohort	United Kingdom	12 mo		23.8 (1.8)	23.0 (1.6)	237	0.4 (0.1)^b^	258	0.4 (0.1)^b^	cm	US	237	2.7 (0.5)^b^	258	2.8 (0.6)^b^	cm	US
Brei et al (2015)^40^	Cohort	Germany	12 mo		16.6 (1.5)	17.0 (1.4)	80	30.4 (14.4)^b^	76	26.4 (11.9)^b^	mm^2^	US	79	18.0 (5.8)^b^	76	17.5 (5.9)^b^	mm^2^	US
Barros et al (2016)^42^	Cohort	Brazil	6 mo		9237 (952)^a^	8910 (110)^a^	11	56.1 (10.8)^b^	5	48.0 (10.2)^b^	mm^3^	US	11	15.6 (1.6)^b^	5	15.6 (1.7)^b^	mm^3^	US
					8508 (1.136)^a^	8547 (220)^a^	19	56.4 (10.8)^b^	18	53.1 (10.1)^b^			19	15.9 (1.6)^b^	18	15.1 (1.9)^b^		
					8358 (551)^a^	8781 (888)^a^	11	56.7 (10.5)^b^	12	58.2 (10.7)^b^			11	15.5 (1.6)^b^	12	14.9 (1.9)^b^		
de Fluiter et al (2020)^43^	Cohort	NL	6 mo		7.35 (0.79)^a^	7.92 (0.85)^a^	173	0.41 (0.12)^b^	228	0.41 (0.11)^b^	cm	US	173	2.28 (0.58)^b^	228	2.33 (0.64)^b^	cm	US
			9 mo		8.49 (0.87)^a^	9.14 (0.99)^a^	173	0.38 (0.10)^b^	228	0.37 (0.10)^b^			173	2.46 (0.70)^b^	228	2.46 (0.67)^b^		
			1 y		9.37 (0.95)^a^	10.01(1.12)^a^	173	0.34 (0.09)^b^	228	0.34 (0.10)^b^			173	2.44 (0.64)^b^	228	2.46 (0.67)^b^		
≥1–2.9 years																		
Holzhauer et al (2009)^44^	Cohort	NL	13.7 mo		16.5(1.1)		104	49.5 (37; 60.8)^d^	108	43.5 (33.3; 58.8)^d^	mm^2^	US	104	28 (20; 32)^d^	108	24 (20; 30.8)^d^	mm^2^	US
			25.3 mo		15.5(2.6)		120	48.7 (38; 63)^d^	107	42.3 (34.2; 57.4)^d^			120	37.2 (30.9; 46.4)^d^	107	36.9 (29; 45.8)^d^		
Brei et al (2018)^45^	Cohort	Germany	2 y		16.0 (1.3)	16.6 (1.3)	48	21.5 (12.6)^b^	63	16.6 (9)^b^	mm^2^	US	48	24.4 (6.8)^b^	63	23.1 (8.0)^b^	mm^2^	US
de Fluiter et al (2020)^43^	Cohort	NL	1.5 y		10.86 (1.04)^a^	11.46 (1.26)^a^	173	0.31 (0.09)^b^	228	0.32 (0.10)^b^	cm	US	173	2.29 (0.61)^b^	228	2.32 (0.65)^b^	cm	US
			24 mo		12.17 (1.17)^a^	12.76 (1.45)^a^	173	0.32 (0.09)^b^	228	0.32 (0.10)^b^			173	2.15 (0.57)^b^	228	2.15 (0.58)^b^		
3 to 6 years																		
Brei et al (2018)^45^	Cohort	Germany	3 y		15.4 (1.0)	15.9 (1.2)	50	23.8 (13.7)^b^	53	15.7 (8.6)^b^	mm^2^	US	49	34.6 (11.7)^b^	53	30.8 (10.5)^b^	mm^2^	US
			4 y		15.2 (1.2)	15.7 (1.1)	42	24.9 (13.1)^b^	51	15.9 (9.8)^b^			43	44.3 (13.8)^b^	51	37.5 (13.4)^b^		
			5 y		15.0 (1.3)	15.7 (1.3)	47	24.5 (14.4)^b^	50	17.1 (10.2)^b^			47	51.7 (14.2)^b^	49	45.2 (13.6)^b^		
Karlsson et al (2013)^46^	Cohort	Sweeden	5 y		14.6 (1.3)	15.4 (1.7)	18	0.71 (0.22)^b^	30	0.71 (0.35)^b^	L	MRI	18	0.10 (0.04)^b^	30	0.17 (0.08)^b^	L	MRI
Sadananthan et al (2019)^47^	Cohort	Singapore	4.5 y		15.6 (1.9)	15.8 (1.9)	166	623.9 (420.4)^b^	150	494.9 (346.6)^b^	mL	MRI	166	186.2 (81.7)^b^	150	195.4 (72.1)^b^	mL	MRI
Durmus et al (2014)^48^	Cohort	NL	6 (5.7; 7.4)	6 (5.7; 7.4)	15.9 (13.9; 20.3)	15.9 (14.0; 19.5)	2633	44 (21.0; 113.0)^d^	2610	35 (17.0; 35.6)^d^	mm^2^	US	2633	58 (26.0; 174.0)^d^	2610	41 (19.0; 129.7)^d^	mm^2^	US
≥6 to 10 years																		
Nagy et al (1997)^49^	Cross-sectional	USA	7.3 (1.7)	7.6 (1.6	18.6 (4.2)	19 (5.6)	23	59 (77)^b^	20	42 (83)^b^	cm^2^	CT	23	20 (17)^b^	20	22 (23)^b^	cm^3^	CT
			7.6 (1.4)	8.2 (1.5)	18.8 (2.7)	17.9 (3.1)	8	84 (70)^b^	23	56 (72)^b^			8	32 (23)^b^	23	24 (20)^b^		
Herd et al (2001)^50^	Cohort	USA	8.2 (1.4)	8.4 (1.3)	20.6 (5.7)		29	119.8 (103.5)^b^	33	91.6 (112.9)^b^	cm^2^	CT	29	29.8 (18.5)^b^	33	35.9 (30.2)^b^	cm^2^	CT
			8.5 (1.2)	8.6 (1.2)	19.3 (4.1)		14	143.3 (113.3)^b^	25	94.5 (79.0)^b^			14	47 (31.6)^b^	25	33.4 (15.7)^b^		
Arfai et al (2002)^51^	Cross-sectional	USA	8.1(1.2)	8.0 (1.2)	17.59 (3.29)	17.61 (3.12)^a^	31	4361 (4582)^b^	31	3325 (4134)^b^	mm^2^	CT	31	803 (765)^b^	31	720 (646)^b^	mm^2^	CT
Huang et al (2002)^52^	Cross-sectional	USA	8.4 (2)	7.9 (1.5)	19.3 (4.8)	20.3 (5.1)	22	100.9 (93.8)^b^	20	105.2 (130.4)^b^	cm^2^	CT	22	26.3 (14.8)^b^	20	36.7 (31.7)^b^	cm^2^	CT
			8.4 (1.5)	8.4 (1.6)	18.6 (3.6)	18.8 (4)	17	108.3 (79.5)^b^	18	103.1 (99)^b^			17	34.8 (19.9)^b^	18	31.7 (20.8)^b^		
Liem et al (2009)^53^	Cross-sectional	United Kingdom	6.8 (6.0; 7.9)	6.7 (6.3; 7.5)	15.7 (13.5; 20.)^a^	16.3(14.6; 19.4)^a^	17	68.2 (34; 169)^d^	14	60.4 (18; 132)^d^	cm^3^	CT	17	21.6 (11.6; 35.4)^d^	14	26 (18.3; 37.7)^d^	cm^3^	CT
Satake et al (2010)^54^	Cross-sectional	Japan	7.9 (1.3)	7.9 (1.4)	15.6 (2.5)	16.0 (1.9)	16	44.9 (30)^b^	20	31.6 (20.6)^b^	cm^2^	CT	16	15.2 (5.5)^b^	20	12.1 (3.7)^b^	cm^2^	CT
Casazza et al (2011)^55^	Cross-sectional	USA	9.3 (0)	9.7 (0.1)	−0.04 (0.1)^e^	−0.04 (0.1)^e^	132	110.1 (8.2)^b^	146	78.6 (6.3)^b^	cm^2^	CT	132	34 (2.2)^b^	146	32.9 (2.2)^b^	cm^2^	CT
Halvorsen et al (2015)^56^	Cohort	USA	7 (1.2)	7 (1.2)	17 (2.5)	18 (3.7)	43	35.3 (26.2)^b^	44	40.3 (42)^b^	cm^2^	CT	43	11.3 (5.3)^b^	44	12.6 (7.3)^b^	cm^2^	CT
Malpique et al (2018)^57^	Cohort	Spain	8.2 (0.2)	8.7 (0.2)	0 (0.3)^e^	0 (0.3)^e^	21	45 (7)^b^	20	36 (6)^b^	cm^2^	MRI	21	15 (1)^b^	20	15 (1)^b^	cm^2^	MRI

a Weight in kilograms.

b Values are mean (SD).

c Weight in grams.

d Values are median (IQR).

e BMI *z*-score.

Abbreviations: BMI, body mass index (kg/m^2^); CT, computed tomography; MRI, magnetic resonance imaging; NL, Netherlands; SDS, standard deviation score; US, ultrasonography.

### Measurement Techniques

In 8 studies (40%),^49–56^ CT was the imaging method used for assessing abdominal fat, with samples limited to children aged 6–10 years. Four studies (20%) utilized MRI with segmentation guided by software,^41^^,^^46^^,^^47^^,^^57^ while 8 studies (40%) used ultrasonography.^38–40^^,^^42–45^^,^^48^ Among the ultrasonography studies, 3 measured VAT using a retroperitoneal plane.^38^^,^^39^^,^^43^ A total of 5 studies (25%) measured preperitoneal fat as an indicator of VAT.^40^^,^^42^^,^^44^^,^^45^^,^^48^ Two studies simultaneously measured superficial and deep SAAT.^41^^,^^47^

### Association of Sex With SAAT

**Figure 2.**
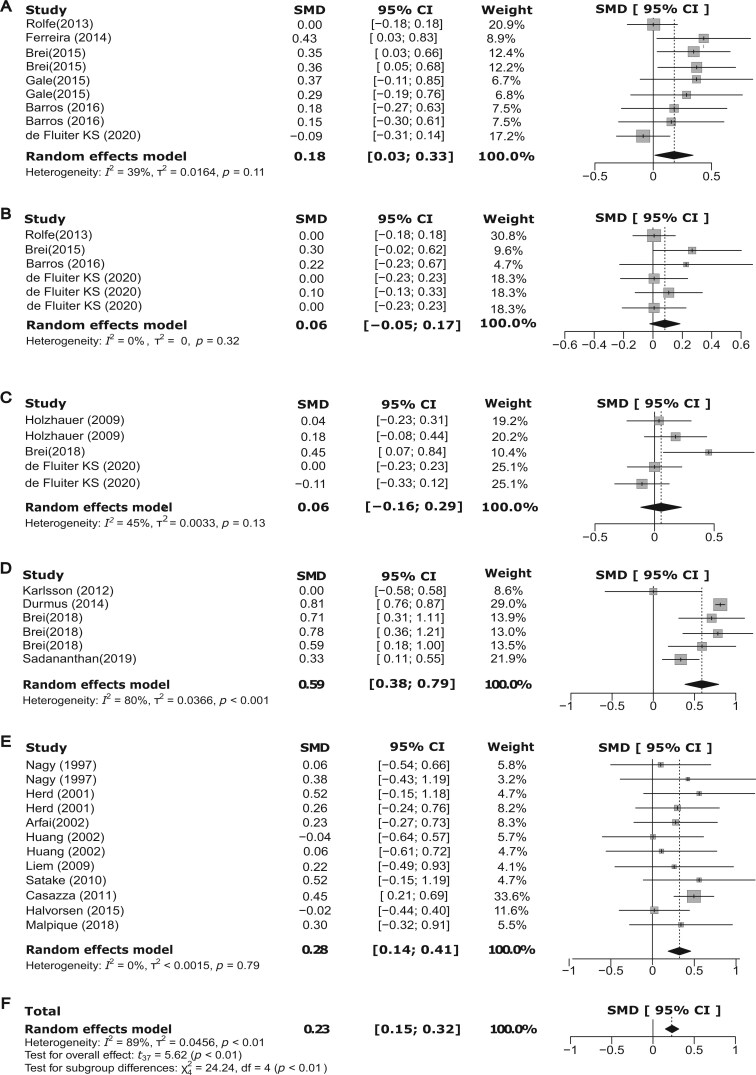
Forest Plots Showing the Effect of Sex on SAAT in Normal-Weight Children Aged Between 0 and 10 Years. Individual plots represent 0 to 4 months (A), 6 to 12 months (B), older than 1 to 2.9 years (C), 3 to 6 years (D), older than 6 to 10 years (E), and the cumulative overall effect (F). Results are presented as the SMD and its 95% CI. Abbreviations: SAAT, subcutaneous abdominal adipose tissue; SMD, standardized mean difference

The effect of sex on SAAT was assessed in 5394 girls and 5568 boys. The overall impact of sex on SAAT was moderate, with girls showing higher levels compared with boys (SDM = 0.23; 95% CI: 0.15–0.32; *I^2^* = 89%) (**Figure 2F**). SAAT was significantly higher in girls aged 0 to 4 months (SMD = 0.18; 95% CI: 0.03–0.33; *I^2^* = 39%) (**Figure 2A**). A moderate effect was observed in girls aged 3 to 6 years (SMD = 0.59; 95% CI: 0.38–0.79; *I^2^* = 80%) (**Figure 2D**) and in those aged over 6 to 10 years (SMD = 0.28; 95% CI: 0.14–0.41; *I^2^* = 0%) (**Figure 2E**). In contrast, no significant differences were observed in children aged over 4 months to 1 year (SMD = 0.06; 95% CI: –0.05 to 0.17; *I^2^* = 0%) (**Figure 2B**) or 1 to 2.9 years (SMD = 0.06; 95% CI: –0.16 to 0.29; *I^2^* = 45%) (**Figure 2C**). Only the group aged 3 to 6 years had considerable heterogeneity (*I^2^* = 80%).

### Association of Sex With VAT

**Figure 3.**
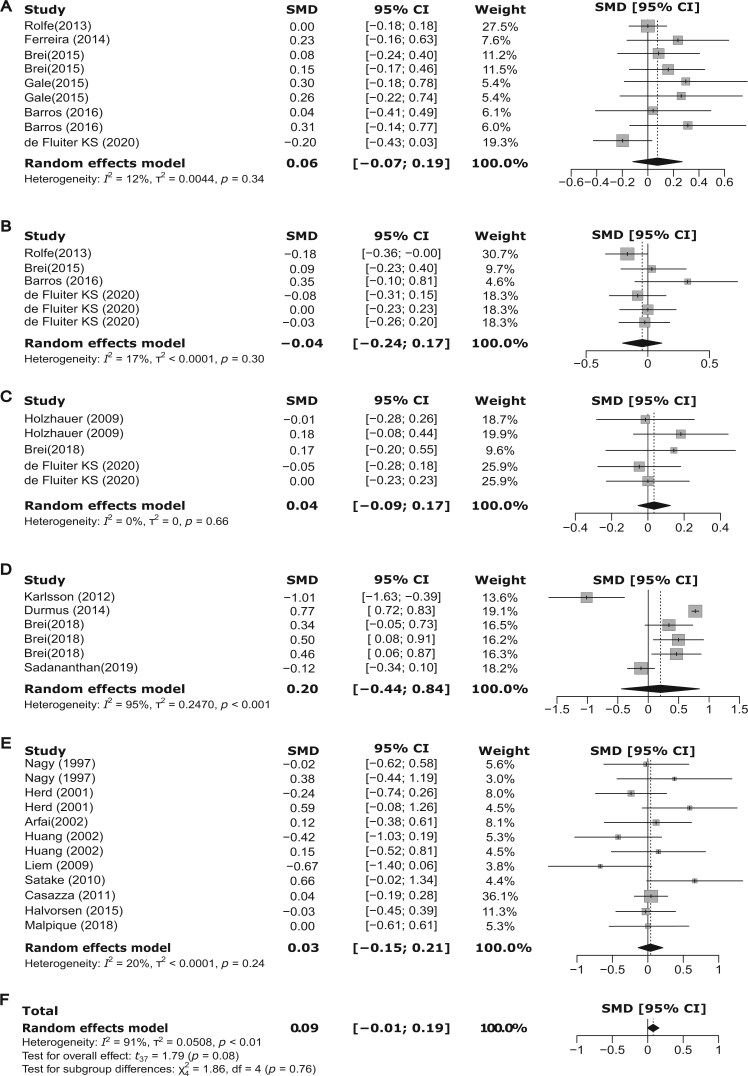
Forest Plots Showing the Effect of Sex on VAT in Normal-Weight Children Aged Between 0 and 10 Years. Individual plots represent 0 to 4 months (A), 6 to 12 months (B), older than 1 to 2.9 years (C), 3 to 6 years (D), older than 6 to 10 years (E), and the cumulative overall effect (F). Results are presented as the SMD and its 95% CI. Abbreviations: SMD, standardized mean difference; VAT, visceral adipose tissue

The effect of sex on VAT was assessed in 5382 girls and 5567 boys. Overall, no significant association was found between sex and VAT (SMD = 0.09; 95% CI: –0.01 to 0.19; *I^2^* = 91%) (**Figure 3F**). This result was consistent across all age groups: 0 to 4 months (SMD = 0.06; 95% CI: –0.07 to 0.19; *I^2^* = 12%) (**Figure 3A**), 6 months to 1 year (SMD = –0.04; 95% CI: –0.24 to 0.17; *I^2^* = 17%) (**Figure 3B**), 1 to 2.9 years (SMD = 0.04; 95% CI: –0.09 to 0.17; *I^2^* = 0%) (**Figure 3C**), 3 to 6 years (SMD = 0.20; 95% CI: –0.44 to 0.84; *I^2^* = 95%) (**Figure 3D**), and over 6 to 10 years (SMD = 0.03; 95% CI: –0.15 to 0.21; *I^2^* = 20%) (**Figure 3E**).Given the considerable heterogeneity observed, these findings should be interpreted with caution.

### Sensitivity Analysis

High heterogeneity was observed in the 3- to 6-year-old group for both SAAT and VAT. In the 0- to 4-months group, exclusion of the study by de Fluiter et al^43^ substantially reduced heterogeneity in SAAT estimates (SMD = 0.22; 95% CI: 0.08–0.35; *I^2^* = 18.8%), while still indicating significantly higher SAAT in girls. For other groups, omitting a single study did not change the result for SAAT (Table S3). In the 3- to 6-year-old age group, removing the study by Durmuş et al^48^ reduced heterogeneity in the SAAT analysis (SMD = 0.50; 95% CI: 0.37–0.63; *I^2^* = 27.5%), while maintaining the direction and significance of the effect, with higher SAAT observed in girls. For VAT, omitting Fluiter et al^43^ in the group aged 0 to 3 months resulted in a significantly higher VAT in girls (SMD = 0.1866; 95% CI: 0.0031–0.204; *I^2^* = 0.0%). The sensitivity analysis in the other groups did not show changes in the association with VAT (Table S4).

### Subgroup Analyses of SAAT and VAT

The subgroup analysis of SAAT did not show differences by design (cohort, cross-sectional), measurement technique (MRI, CT, ultrasound), or quality (Table S5). The reports from Asia (2 studies) did not show the effect of sex over SAAT (SMD = 0.3509; 95% CI: –0.3475 to 1.0493; *I^2^* = 0%). Otherwise, we found higher VAT in girls when it was measured by ultrasonography (SMD = 0.1280; 95% CI: 0.0158–0.2403; *I*^2^ = 94%) or when VAT was estimated using preperitoneal adipose tissue (SMD = 0.2338; 95% CI: 0.0776–0.390; *I^2^* = 91.7%) (Table S6).

### Meta-regression

The univariate meta-regression analysis for SAAT demonstrated that age was significantly associated with SMD (β =  0.029, *P* = .0318), accounting for 22.83% of the heterogeneity. This suggests that older children exhibit larger sex differences in SAAT. In contrast, measurement techniques (*P* = .932), study design (cohort vs cross-sectional; *P* = .594), geographic region (*P* = .8872), and study quality (low vs high; *P* = .365) did not significantly explain heterogeneity. However, the sample size was significantly associated with the SMD (β = .0001, *P* = .001), explaining 46.19% of the variability. Overall, the analyses showed that age and sample size were significant contributors to heterogeneity in the effect by sex for SAAT in children (Table S7).

For VAT, the sample size was the only significant contributor to heterogeneity (β = 0.0001, *P* < .001), accounting for 80.89% of the variability. Age (*P* = .673), measurement technique (*P* = .453), study design (*P* = .806), geographic region (*P* = .801), and study quality (*P* = .837) did not significantly explain heterogeneity in VAT (Table S7).

### Publication Bias

The meta-analysis showed symmetrical funnel plots and nonsignificant Egger’s tests (*P* > .05) for SAAT in the 6-months to 1-year, 3- to 6-years, and over 6- to 10-years groups, and for VAT in the 3- to 6-years and over 6- to 10-years groups, suggesting no statistical evidence of small-study effects. In contrast, significant Egger’s test results (*P* < .05) were observed for both SAAT and VAT in the 0- to 4-months group, and for VAT in the 6-months to 1-year group (**Figures S1** and **S2**). While these results may indicate potential small-study effects, they should be interpreted with caution due to the limited number of studies in these subgroups. Publication bias could not be assessed in the group aged more than 1 to 2.9 years due to an insufficient number of studies (<6).

## DISCUSSION

This systematic review and meta-analysis found that girls accumulate greater SAAT than boys, particularly from the age of 3 years. No significant sex differences in VAT were observed during the prepubertal stage; however, heterogeneity in the measurement techniques used to assess VAT may have masked potential small differences.

The biological mechanisms for sex differences in SAAT (higher in girls) at the prepubertal stage remain unclear. Sex- and growth-related hormones might be the first factors to consider supporting sex differences. However, at the onset of the prepubertal stage, circulating levels of estradiol and testosterone are low, and the limited existing evidence on their association with adiposity markers in children remains inconclusive. Garnett et al^58^ reported that girls aged 7–8 years exhibited greater trunk and abdominal fat, along with higher levels of insulin-like growth factor 1 (IGF-I), estradiol, and testosterone compared with boys. Although these hormones were significantly correlated with abdominal fat, the associations were weak (*r* = 0.139–0.178). However, when analyzed by sex, estradiol was significantly associated with abdominal fat only in boys, showing a stronger correlation (*r* = 0.33). A major limitation of this study was the inability to distinguish between SAAT and VAT. In contrast, a report in Chilean girls aged 7 years found no association between estradiol and central adiposity,^59^ although this finding may be attributed to limited power, as it involved a small female-only subsample (*n* = 107) of a larger cohort (*n* = 1190). These studies highlight inconsistencies in the existing literature and underscore the need for more studies to clarify the hormonal influences on early sex differences in abdominal fat distribution.

Our finding of overall higher SAAT in girls is consistent, to some extent, with reports indicating that subcutaneous fat is a major source of leptin secretion into the bloodstream, in contrast to visceral fat.^60^ Correspondingly, higher leptin levels have been observed in prepubertal girls compared with boys. A recent meta-analysis of 21 studies in children aged 0–10 years reported a pooled mean difference in serum leptin of 1.72 ng/mL (95% CI: 1.25–2.19 ng/mL), with this sexual dimorphism becoming evident from age 3 years.^19^ Similarly, we observed that sexual dimorphism in SAAT became statistically significant in the 3- to 6-years age group. The implications of this age cutoff for sex-specific differences remain unclear based on the current literature. This age cutoff relatively coincides with a range of 5–6 years old, which has been reported as the life stage from which an adiposity rebound starts, a critical stage for adiposity development.^61^ The adiposity rebound is characterized by an initial rapid increase in BMI during the first year of life, followed by a gradual decline and reaching its nadir at approximately 5 to 6 years of age. Subsequently, BMI increases progressively throughout childhood, signaling the onset of the adiposity-rebound phase.^62^ However, we found no reports describing sex-specific differences in the timing or magnitude of adiposity rebound or in regional fat redistribution during the transition from stable to increasing BMI in childhood. Future studies are needed to address this gap and to explore potential links with the patterns identified in this meta-analysis.

Elucidating these differences may require investigating the underlying biological mechanisms of fat depot development. Adipocytes from VAT and SAAT differ in their developmental origin,^63^ gene expression,^64^^,^^65^ proliferating potential,^63^^,^^66^ metabolism, and secretion of adipokines profile.^67^ Sex-based variations in the distribution of these depots have also been linked to differences in the tissue microenvironment and extracellular matrix composition,^68^ cellular differences in enzymatic activity,^69^ and thus as the expression of estrogenic (α, β) receptors.^70^ These factors may help explain the greater efficiency in free fatty acid uptake by SAAT observed in women compared with men without obesity.^71^ Although the genesis of these molecular differences remains incompletely understood, it has been proposed that inherent inequality in sex chromosomes (XX vs XY) between males and females and epigenetic modifications, such as methylation of cytosines and histone modifications, may be involved.^21^^,^^72^

In lean individuals, SAAT expansion exhibits sex-specific patterns: women predominantly show adipocyte hyperplasia, while men more commonly exhibit adipocyte hypertrophy.^73^ These differences may contribute to sexual dimorphism in metabolic risk, as hypertrophic expansion is more strongly associated with insulin resistance.^74^ In women with obesity, SAAT volume has been positively associated with proinflammatory markers such as circulating leukocytes and interleukin-6 (IL-6), a relationship not observed in men.^75^ In adults, SAAT has been linked to higher risks of type 2 diabetes and coronary artery disease, although these associations are attenuated after adjustment for BMI or waist circumference (WC).^12^^,^^76^ In pediatric populations, SAAT has shown independent associations with metabolic syndrome components, even after BMI adjustment. Maffeis et al^16^ reported that higher SAAT volumes were associated with lower insulin resistance in prepubertal children with overweight or moderate obesity, suggesting a potential early-life protective role. The metabolic duality of SAAT can be attributed to its anatomical division by Scarpa's fascia, which generates 2 functionally distinct layers. On one hand, deep SAAT that shows higher saturated fatty acid content^77^ and inflammatory profile gene expression (eg, IL-6, Monocyte Chemotactic Protein-1, resistin),^17^^,^^78^ in contrast to superficial SAAT, presents protective features such as higher expression of adipokines such as leptin^79^ and adiponectin.^80^ However, the extent to which these distinct SAAT layers exhibit sex-specific functional properties in early life remains insufficiently characterized.

We observed no sex differences in VAT, consistent with reports indicating that sexual dimorphism in visceral fat typically emerges during adolescence.^81^^,^^82^ However, the high heterogeneity in the overall model (*I^2^* = 91%) limits the reliability of the pooled estimates. Although subgroup and meta-regression analyses were performed, they did not fully explain the variability across studies. This suggests that differences in study design and measurement protocols may have influenced the effect estimates, highlighting the need for cautious interpretation. VAT was measured using imaging techniques such as MRI, CT, and ultrasound across all included studies. It remains possible that variations in imaging protocols, anatomical landmarks, or segmentation criteria contributed to residual heterogeneity, potentially masking subtle sex-specific differences in VAT during the prepubertal stage. VAT can also be measured using other techniques, such as DXA, bioelectrical impedance analysis, and anthropometric ratios such as waist-to-hip ratio, waist-to-height ratio, and WC.^83^ It is important to note that accuracy can vary depending on the technique used, and all methods are susceptible to errors inherent in either the measurement technique or the evaluator. MRI and CT are considered the gold standards for measuring VAT and SAAT in children.^84^ Among the selected studies, 60% used one of these techniques. Although MRI can accurately quantify abdominal fat distribution through multi-slice imaging, its application in pediatric studies is often limited by several factors: the need for deep sedation, high costs, lengthy scan times, limited accessibility, and excessive noise levels.^85^^,^^86^ Computed tomography was primarily restricted to children over 6 years in our meta-analysis, since, although offering excellent contrast resolution and soft-tissue differentiation, the technique has inherent limitations of radiation exposure and frequent need for sedation.^87^^,^^88^ Although MRI and CT are the most reliable techniques for determining SAAT and VAT, they are considered unsuitable for epidemiological and clinical studies, particularly in young children.^89^^,^^90^ This may account for the more frequent use of ultrasound in studies involving children under 6 years of age, given its noninvasive nature, absence of ionizing radiation, and relatively lower cost compared with other imaging modalities for estimating abdominal adiposity.^91^

Ultrasonography was used in 40% of the included studies to assess abdominal fat distribution. Although it is widely accessible and noninvasive, its operator dependence and limited reproducibility, particularly in the assessment of VAT, may compromise its reliability in detecting subtle differences.^92^ De Lucia Rolfe et al^38^ found that the accuracy of ultrasound measurements for adipose tissue is depot-dependent when compared with MRI, with stronger correlations for SAAT (*r* = 0.71) than for VAT (*r* = 0.48). Similarly, studies in pediatric populations, such as those by Mook-Kanamori et al,^93^ reported high correlations for SAAT (*r* = 0.94–0.97) and moderate correlations for VAT (*r* = 0.75–0.84) in nonobese children using MRI. Koot et al^94^ also observed a lower correlation for VAT (*r* = 0.60) in obese cohorts when compared with CT. These studies suggest that ultrasound is a suitable method for estimating abdominal adipose tissue in children; however, it may lack the sensitivity to detect sex differences, particularly within the VAT compartment.

In the subgroup analysis of studies that measured preperitoneal fat using ultrasonography, we found that VAT was higher in girls (SMD = 0.15; 95% CI: 0.03–0.28; *I^2^* = 93.5%). Some studies suggest that preperitoneal fat assessed by ultrasound is a useful alternative for estimating VAT in pediatric populations.^93^^,^^95^ Although CT and MRI are considered the best techniques for evaluating abdominal adiposity, several studies have shown that ultrasound, when performed using standardized protocols, can provide valid and reliable estimates of both VAT and SAAT. Reported correlations between ultrasound and MRI assessments of VAT in adult populations range from 0.67 to 0.91, with the highest correlation (*r* = 0.96) observed when MRI measurements were taken at the L2–L3 vertebral level.^96^ A strong correlation has also been reported between ultrasound and CT measurements of SAAT thickness (ρ = 0.93), although detecting small variations in SAAT thickness with ultrasound may be less accurate than with CT.^97^ However, as mentioned earlier, validation studies have shown only moderate correlations between ultrasonography-based VAT measurements and gold-standard techniques in prepubertal children, whereas stronger correlations have been reported in adolescents and adults.^98^ Factors such as smaller VAT volumes and bowel peristalsis in younger children may reduce the accuracy of ultrasound assessments in the pediatric population.^38^

While significant heterogeneity was observed for SAAT and VAT, the sex differences in SAAT remained significant across sensitivity analyses despite varying levels of heterogeneity (*I^2^* =18.8%–48.2%). Meta-regression results (with age explaining 22.83% of SAAT heterogeneity) suggest that these variations reflect a biological difference rather than a methodological bias. For VAT, heterogeneity across techniques (particularly ultrasound; *I^2^* = 94%) suggests technique-dependent detection sensitivity, requiring cautious interpretation of VAT-related findings, especially for ultrasound measurements. Nevertheless, the overall null finding for sex differences remained consistent across most analyses. Importantly, neither study quality nor design significantly contributed to heterogeneity, suggesting that our conclusions are reasonably well supported.

Our finding that girls accumulate greater SAAT than boys after age 3 years, without corresponding differences in VAT, may have important implications for interpreting epidemiological associations between VAT and anthropometric indicators of abdominal fat in children under 10 years, such as WC, which represents a composite indicator of both fat depots. Waist circumference is a weak predictor of VAT between the ages of 4 and 10 years when compared with CT measurements.^99^ Brambilla et al^100^ reported that WC explained only 64.8% of the VAT and 80.4% of SAAT variability in children. Karlsson et al^46^ found in preschool-aged children that WC correlated more strongly with trunk fat mass (*r* = 0.86) than with VAT (*r* = 0.43). The low sensitivity of WC for estimating VAT at early ages may be attributed to sex differences in SAAT depots. These findings could be related to studies reporting sex-based differences in the WC–cardiometabolic risk association. For instance, Hitze et al^101^ found in children and adolescents (age 6.1–19.9 years) significant age/puberty-adjusted correlations between WC and triglyceride levels in girls but not in boys. In contrast, WC measurements in boys significantly correlated with low-density-lipoprotein (LDL) cholesterol levels and the Homeostatic Model Assessment of Insulin Resistance (HOMA-IR). With regard to hemodynamic variables of cardiometabolic risk, in Korean adolescents aged 12–16 years, WC was significantly related to stroke volume, cardiac output, systolic blood pressure, pulse pressure, and vascular function only in boys.^102^ In agreement with the above finding, Samouda et al^103^ reported in youth (age 12–17 years) affected by overweight/obesity that WC was a suitable surrogate of VAT in boys but not completely in girls with overweight or obesity, since, in females, the prediction models for VAT were improved by subtracting SAAT. Further studies conducted in prepubertal populations that discriminate between the type of abdominal fat and cardiometabolic risk factors in the function of sex could help disclose better-characterized patterns for the association between abdominal adiposity and cardiometabolic risk at early stages of life.

**Figure 4.**
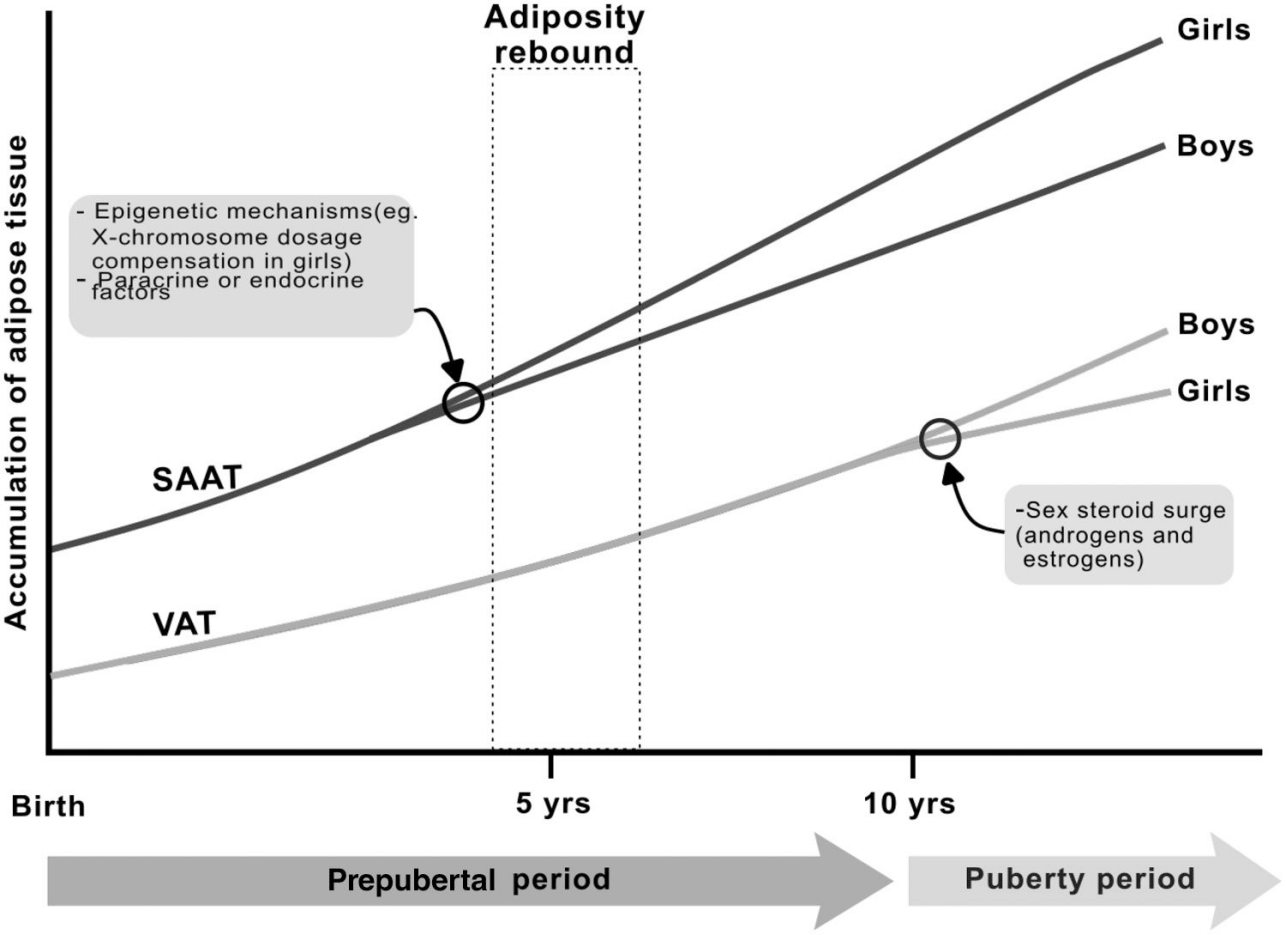
Developmental Model of Sexual Dimorphism in Abdominal Fat Distribution. Sex differences in SAAT emerge around adiposity rebound (∼5–6 years), possibly driven by epigenetic mechanisms such as X-chromosome dosage compensation. During puberty, rising sex hormones amplify these differences, leading to greater VAT accumulation in boys. Abbreviations: SAAT, subcutaneous abdominal adipose tissue; VAT, visceral adipose tissue; yrs, years

Our findings suggest that the developmental programming of SAAT may begin earlier in girls, while sex-specific differences in VAT appear to emerge later, likely after puberty. Based on these observations, we propose a developmental model in which sexual dimorphism in SAAT arises in early childhood, around the age of 3 years, preceding the onset of adiposity rebound (Figure 4). This early divergence may be driven by sex-specific biological mechanisms, including epigenetic regulation potentially related to X-chromosome dosage compensation in girls, as well as other epigenetic influences or unidentified endocrine and paracrine signaling pathways. These initial differences in fat distribution may establish the foundation for more pronounced divergence during puberty, when increasing levels of sex steroid hormones, particularly estrogens and androgens, further shape sex-specific patterns of fat accumulation. This process results in a greater deposition of VAT in boys compared with girls. However, findings related to VAT should be interpreted with caution due to the substantial heterogeneity observed across studies.

### Limitations and Strengths

Our results should be treated with caution because of the following limitations. First, the scope of our review was limited to English-language, full-text publications retrieved from 4 electronic databases. Second, there was considerable heterogeneity among the studies included in this review. This heterogeneity may be due to several factors, including specific background characteristics of the pediatric populations and different measurement techniques used to estimate fat distribution, some of which are not the gold standard for measuring abdominal fat, although sensitivity and subgroup analyses were performed to mitigate this problem. Third, although Egger’s tests and funnel plots indicated no significant publication bias in most age groups, significant results in the 0- to 4-months group (for both SAAT and VAT) and in the 6-months to 1-year group (for VAT) may suggest small-study effects. However, these results should be interpreted with caution, as the limited number of studies in these subgroups reduces the reliability of both Egger’s test and visual assessment of funnel plot asymmetry. This limitation could hide sex-specific differences in VAT during early infancy. While these subgroups represented a small portion of the overall analysis, the potential for biased reporting should be considered when interpreting our findings, particularly in age ranges with limited data. Fourth, most of the study samples meta-analyzed in this review were from North American and European populations; hence, the other Asian, African, and Latin American populations were underrepresented. Fifth, the lack of Tanner stage reporting in many of the included studies limits the ability to definitively exclude early or precocious puberty among participants under 10 years of age. While using an age-based cutoff (≤10 years) increases the likelihood that most participants were prepubertal, the possible inclusion of children undergoing early pubertal transitions cannot be ruled out. It may have introduced bias, particularly in estimating sex-based differences in VAT and SAAT among older children. This limitation should be considered when interpreting the results. Nevertheless, the consistent and statistically robust finding of greater SAAT in girls from as early as 3 years of age—an age at which pubertal onset is biologically implausible—supports the validity of this association and suggests that sex-related differences in abdominal fat distribution may emerge from early-life biological or developmental mechanisms, independent of puberty.

Other strengths of this research include the sex balance of the sample between girls and boys in the different age groups, the consistency of the results, and the fact that the sensitivity analysis did not show that the results were modified by factors such as measurement technique, study design, or geographical region.

## CONCLUSION

Our analysis indicates that sex differences in abdominal adiposity begin to emerge as early as age 3 years, with girls showing significantly greater SAAT deposition compared with boys. In contrast, VAT distribution did not show statistically significant sex-based variation across any age strata, although the high heterogeneity observed limits the interpretability of this finding. The increased SAAT in girls is unlikely to be driven by sex hormone differences, suggesting the involvement of alternative biological mechanisms that indicate the need for further investigation. The observed sex differences in SAAT may have important implications for the interpretation of indirect measures of VAT, such as WC, and their associations with cardiometabolic risk in children.

## Supplementary Material

nuaf143_Supplementary_Data

## Data Availability

All data included in this study are provided in this article and the supplementary material file.
